# Application of machine learning models for predicting risk factors of acute exacerbations in chronic obstructive pulmonary disease

**DOI:** 10.3389/fmed.2026.1804544

**Published:** 2026-05-14

**Authors:** Dapeng Kuang, Jie Min, Huibiao Deng, Yajun Zhao, Yangyang Sun, Jiang Hong

**Affiliations:** 1 Department of Emergency and Critical Care, Shanghai General Hospital, Shanghai Jiao Tong University School of Medicine, Shanghai, China; 2 Clinical Research Center for Mental Disorders, Shanghai Pudong New Area Mental Health Center, School of Medicine, Tongji University, Shanghai, China; 3 Department of Health Management Centre, Zhongshan Hospital, Fudan University, Shanghai, China; 4 Henan Cancer Hospital, Zhengzhou, Henan, China

**Keywords:** acute exacerbations of COPD (AECOPD), chronic obstructive pulmonary disease (COPD), machine learning, risk factors, XGBoost

## Abstract

**Background:**

Chronic obstructive pulmonary disease (COPD) is a chronic respiratory disease characterized by persistent respiratory symptoms and progressive airflow limitation. Acute exacerbations of COPD (AECOPD) are significant causes of hospitalization and death among COPD patients. This study aims to identify risk factors for AECOPD exacerbations and develop a highly accurate and interpretable predictive model using various statistical and machine learning methods.

**Methods:**

We retrospectively analyzed data from 2,102 COPD patients admitted between 1 January 2019 and 31 December 2024. The primary outcome was AECOPD severity, defined as the need for treatment escalation. Initial feature selection was performed using LASSO regression to identify potential risk factors. To validate the model’s effectiveness and explore its superior predictive performance, the dataset was partitioned by time period and proportion: The first 70% of observations in chronological order were used as the training set, with the remaining 30% as the test set. Multiple machine learning algorithms were then employed for model construction and comparison. To enhance model interpretability, we utilized SHapley Additive exPlanations (SHAP) to illustrate the contribution of each variable to the prediction outcomes.

**Results:**

Among the six machine learning models, the extreme gradient boosting (XGBoost) model demonstrated the optimal predictive performance, achieving an area under the receiver operating characteristic curve (AUC) of 0.960 (95% confidence interval (CI): 0.940–0.980) in the training set and 0.824 (95% CI: 0.804–0.844) in the test set. In the test set, the evaluation metrics were as follows: accuracy, sensitivity, specificity, positive predictive value (PPV), and negative predictive value (NPV) were 0.805, 0.65, 0.872, 0.669, and 0.859, respectively. SHAP analysis revealed that creatinine (CREA), neutrophil percentage (NEU%), D-dimer, brain natriuretic peptide (BNP), white blood cell count (WBC), and hypertension (HTN) were important factors influencing the model output.

**Conclusion:**

The XGBoost model developed in this study demonstrates robust performance in predicting AECOPD risk using routinely collected clinical and laboratory data. The integration of SHAP analysis enhances model transparency, supporting its potential utility in clinical risk stratification and early intervention.

## 1 Introduction

Chronic obstructive pulmonary disease (1) is a chronic respiratory disease characterized by persistent respiratory symptoms and progressive airflow limitation (1). As the most prevalent chronic respiratory disease in our country, COPD exhibits a continuously rising prevalence (2, 3). Acute exacerbation of chronic obstructive pulmonary disease (AECOPD), defined as an acute worsening of respiratory symptoms requiring additional treatment, can lead to irreversible declines in lung function and rapid deterioration in health status (4–7, 28). Severe acute exacerbations significantly increase the risk of hospitalization and mortality, thereby imposing a substantial burden on both patients and healthcare systems (8–11). Identifying risk factors that predict the occurrence of AECOPD is crucial for early intervention and prevention (12, 33). While previous studies have identified various factors associated with AECOPD, these investigations often rely on traditional statistical methods, such as univariate and multivariate logistic regression (13). These approaches, however, exhibit limitations when handling high-dimensional data and complex relationships. In recent years, advancements in machine learning have provided powerful tools for analyzing complex medical data (14, 15). These techniques enable more accurate identification of risk factors and the development of high-performance predictive models (16–20). This study aims to leverage a combination of statistical and machine learning methods to identify risk factors for AECOPD and establish a predictive model with high accuracy and interpretability. By integrating data cleaning, feature engineering, and advanced machine learning algorithms, this research seeks to enhance clinical management and prevention strategies for AECOPD.

## 2 Materials and methods

### 2.1 Study population

**Figure 1:**
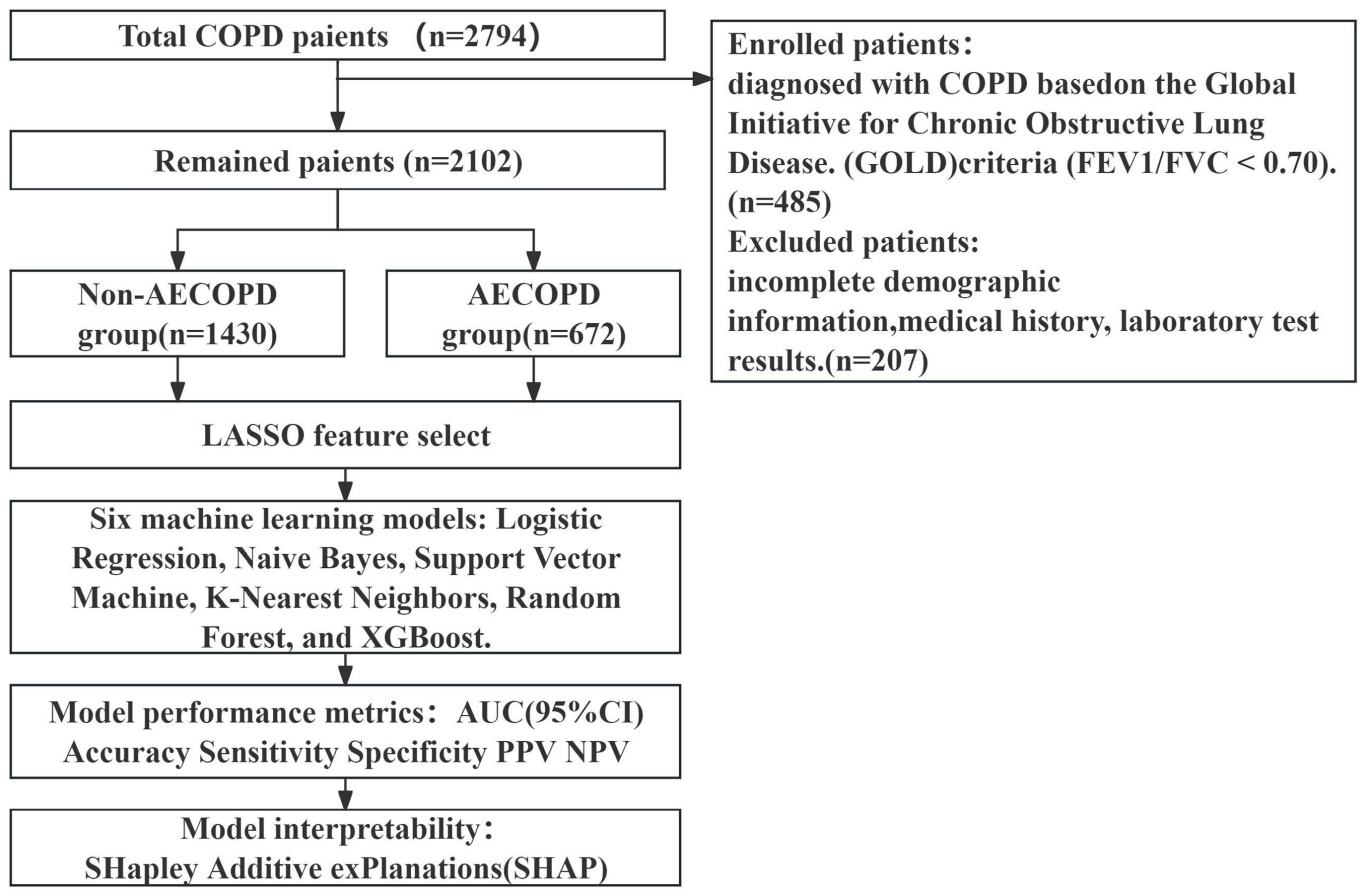
Model development pipeline flowchart.

This study retrospectively enrolled patients at Shanghai General Hospital between 1 January 2019 and 31 December 2024. A total of 2,794 patients were included in the study. The dataset was selected to ensure data quality, consistency in diagnosis, uniformity in clinical practice, and better control over confounding variables. The inclusion criteria were as follows: (1) diagnosis of COPD based on the Global Initiative for Chronic Obstructive Lung Disease (GOLD) criteria (FEV1/FVC < 0.70); 485 non-COPD patients were excluded; (2) availability of complete demographic information, medical history, and laboratory test results; 207 patients were excluded for missing data. After applying the above selection criteria, 2,102 eligible COPD patients were retained, providing a high-quality dataset for risk-factor selection and prediction model development. The detailed inclusion and processing workflow for the study population—including the initial sample size in the original database, reasons for exclusion at each stage, feature selection, model development, model evaluation, and model interpretability analyses—is presented as a flowchart (Figure 1). This retrospective study was approved by the Institutional Review Board of a tertiary general hospital (approval no.: 2021-022-01k) and was conducted in accordance with the ethical principles of the Declaration of Helsinki. All clinical and laboratory data for included patients were de-identified and anonymized prior to statistical analysis; no personally identifiable information was retained, to protect patient privacy. To ensure that the model predicts the future risk of acute exacerbation of COPD (rather than identifying already present or currently worsening exacerbations), all predictor variables were collected strictly prior to the occurrence of the study outcome (i.e., before escalation of therapy was determined). Predictors were obtained from the first set of laboratory tests performed within 24 h of hospital admission, including complete blood count, biochemical panel, coagulation profile, and B-type natriuretic peptide (BNP) measured at emergency or ward admission. Missing values for continuous variables were imputed using the median, and missing categorical variables were imputed using the mode. The Kolmogorov–Smirnov test was used for continuous variables. As continuous variables were all non-normal, the median (interquartile range) was used for description and the Mann–Whitney U test was used to compare differences between groups. Categorical variables were expressed as percentages (%), and Pearson’s chi-squared tests were used to compare differences between groups. All statistical analyses and machine learning model development were performed using Python (version 3.8).

### 2.2 Feature analysis and selection

Pearson’s correlation analysis was used to analyze continuous variables, so as to evaluate the linear correlation strength and direction between two continuous variables. The correlation coefficient r ranges from −1 to 1: r > 0 represents a positive correlation, r < 0 represents a negative correlation, and the closer |r| is to 1, the stronger the linear correlation, while the closer to 0, the weaker the linear correlation. Least absolute shrinkage and selection operator (LASSO) regression was employed for feature selection. The optimal regularization parameter (*λ*) was carefully selected through 5-fold cross-validation. This cross-validation method divides the dataset into five subsets, and the model is trained and evaluated five times, each time using a different subset as the validation set and the remaining four subsets as the training set. This approach helps to obtain a more robust estimate of the model’s performance and select the most appropriate *λ* value.

### 2.3 Model development pipeline

To ensure robust model evaluation, the dataset was partitioned by time period and proportion: The first 70% of observations in chronological order (January 2019–July 2023) were used as the training set, and the remaining 30% (August 2023–December 2024) were used as the testing set. The distribution of the dataset is as follows: Training set: 1471patients (70%), including 982 patients without AECOPD (Class 0) and 489 patients with AECOPD (Class 1). Testing set: 631 patients (30%), including 448 patients without AECOPD (Class 0) and 183 patients with AECOPD (Class 1). The partitioning ensured that the class distribution was consistent between the training and testing sets, minimizing potential bias. Due to class imbalance in the dependent variable, undersampling was applied to resample the data for class balancing. Undersampling was performed only on the training set, while the testing set retained its original distribution. The undersampling procedure was conducted outside the cross-validation loop to avoid data leakage. LASSO was used to select features and reduce dimensions by compressing coefficients. To validate the effectiveness of the predictive model and explore superior performance, six machine learning algorithms were applied, including logistic regression, XGBoost (extreme gradient boosting), random forest, AdaBoost, support vector machine (SVM), k-nearest neighbors (KNN), and Naive Bayes. Each model was trained on the training set to optimize hyperparameters and prevent overfitting. The performance of each model was evaluated using the following metrics: ROC curve and AUC: The receiver operating characteristic (ROC) curve was plotted, and the area under the curve (AUC) was calculated to assess the model’s discriminative ability; Calibration Curve: The calibration curve was used to evaluate the agreement between predicted probabilities and actual probabilities; Decision Curve Analysis (DCA): The DCA curve was generated to assess the net benefit of the model in clinical decision-making; Calibration Curve: The calibration curve was plotted to evaluate the consistency and agreement between the predicted probabilities and the actual observed outcomes; Confusion Matrix: The confusion matrix was constructed to visually and quantitatively display the classification performance of the model, including true positive (TP), true negative (TN), false positive (FP), and false negative (FN); Other Metrics: Accuracy, sensitivity, specificity, positive predictive value (PPV), and negative predictive value (NPV) were calculated to provide a comprehensive evaluation of model performance, with the formulas shown as Equations 1–5.


$$\text{Accuracy}=\frac{\mathrm{TP}+\mathrm{TN}}{\mathrm{TP}+\mathrm{TN}+\mathrm{FP}+\mathrm{FN}}$$ (1)

$$\text{Sensitivity}=\mathrm{TPR}=\frac{\mathrm{TP}}{\mathrm{TP}+\mathrm{FN}}$$ (2)

$$\text{Specificity}=\frac{\mathrm{TN}}{\mathrm{TN}+\mathrm{FP}}$$ (3)

$$\mathrm{PPV}=\frac{\mathrm{TP}}{\mathrm{TP}+\mathrm{FP}}$$ (4)

$$\mathrm{NPV}=\frac{\mathrm{TN}}{\mathrm{TN}+\mathrm{FN}}$$ (5)

To enhance the interpretability of the models, the results of multivariate logistic regression were integrated into the machine learning algorithms. Additionally, SHAP (SHapley Additive exPlanations) values were calculated to quantify the contribution of each feature to the model’s predictions. SHAP values provide a unified measure of feature importance by calculating the marginal contribution of each feature to the model’s output.

## 3 Results

### 3.1 Baseline characteristics

The study included 2,102 hospitalized patients with COPD, who were divided into two groups according to the primary study outcome (whether treatment escalation is required in COPD patients during hospitalization), with 1,471 (69.98%) labeled as Class 0 and 631 (30.01%) as Class 1. Statistical differences were observed between the two groups in NEU%, WBC, NEU#, LYM#, PCT, CREA, GGT, D-dimer, gender, HTN, HD, and CAP (*p* < 0.05), while no significant differences were found in ALP, AST, BNP, DM, HLD, emphysema, and pulmonary bullae (*p* > 0.05). Table 1 shows the comprehensive demographic and clinical information of the 2,102 patients.

**Table 1:** Demographic characteristics of patients and examination pairs in different groups.

Variables	Total (*n* = 2,102)	Non-AECOPD (*n* = 1,430)	AECOPD (*n* = 672)	Statistic	*p*
NEU%, Mean ± SD	68.84 ± 12.27	67.08 ± 11.99	72.59 ± 12.01	t = −9.81	<0.001
WBC, M (Q₁, Q₃)	7.06 (5.60, 8.90)	7.00 (5.49, 8.90)	7.41 (5.77, 9.41)	Z = −3.79	<0.001
NEU#, M (Q₁, Q₃)	4.77 (3.45, 6.35)	4.56 (3.32, 5.87)	5.34 (3.82, 7.38)	Z = −7.11	<0.001
LYM#, M (Q₁, Q₃)	1.36 (0.96, 1.75)	1.42 (1.03, 1.77)	1. 19 (0.86, 1.62)	Z = −6.10	<0.001
PCT, M (Q₁, Q₃)	0. 17 (0.13, 0.21)	0. 17 (0.15, 0.22)	0. 14 (0.07, 0. 19)	Z = − 12.08	<0.001
ALP, M (Q₁, Q₃)	72.30 (60.30, 87.63)	72.60 (60.20, 88. 10)	71.80 (60.55, 86.89)	Z = −0.15	0.879
AST, M (Q₁, Q₃)	20.96 (16.94, 26.39)	21.00 (16.67, 26.80)	20.72 (17.26, 26.00)	Z = −0.56	0.578
CREA, M (Q₁, Q₃)	83.05 (66.30, 143.68)	77.00 (63.92, 94.88)	164.02 (77.92, 442.54)	Z = − 16.42	<0.001
GGT, M (Q₁, Q₃)	28.45 (19.86, 41.55)	27.20 (18.33, 40.09)	29.82 (23.89, 44.41)	Z = −5.72	<0.001
D-dimer, M (Q₁, Q₃)	0.65 (0.35, 1.55)	0.70 (0.37, 1.76)	0.59 (0.33, 1.28)	Z = −3.56	<0.001
BNP, M (Q₁, Q₃)	76.00 (37.00, 178.00)	75.00 (37.00, 179.75)	77.50 (39.00, 175.00)	Z = −0.25	0.804
Gender, *n* (%)				χ^2^ = 4. 11	0.043
0	230 (10.94)	170 (11.89)	60 (8.93)		
1	1872 (89.06)	1,260 (88. 11)	612 (91.07)		
HTN, *n* (%)				χ^2^ = 4.98	0.026
0	1,089 (51.81)	717 (50. 14)	372 (55.36)		
1	1,013 (48. 19)	713 (49.86)	300 (44.64)		
DM, *n* (%)				χ^2^ = 0.34	0.560
0	1721 (81.87)	1,166 (81.54)	555 (82.59)		
1	381 (18. 13)	264 (18.46)	117 (17.41)		
HLD, *n* (%)				χ^2^ = 2.60	0.107
0	2051 (97.57)	1,390 (97.20)	661 (98.36)		
1	51 (2.43)	40 (2.80)	11 (1.64)		
HD, *n* (%)				χ^2^ = 9.80	0.002
0	1834 (87.25)	1,270 (88.81)	564 (83.93)		
1	268 (12.75)	160 (11. 19)	108 (16.07)		
Emphysema, *n* (%)				χ^2^ = 0.30	0.587
0	1805 (85.87)	1,232 (86.15)	573 (85.27)		
1	297 (14. 13)	198 (13.85)	99 (14.73)		
Pulmonary bullae, *n* (%)				χ^2^ = 0. 11	0.745
0	2040 (97.05)	1,389 (97.13)	651 (96.88)		
1	62 (2.95)	41 (2.87)	21 (3.12)		
CAP, *n* (%)				χ^2^ = 10.83	<0.001
0	1795 (85.39)	1,246 (87.13)	549 (81.70)		
1	307 (14.61)	184 (12.87)	123 (18.30)		

### 3.2 Feature analysis and LASSO select

**Figure 2:**
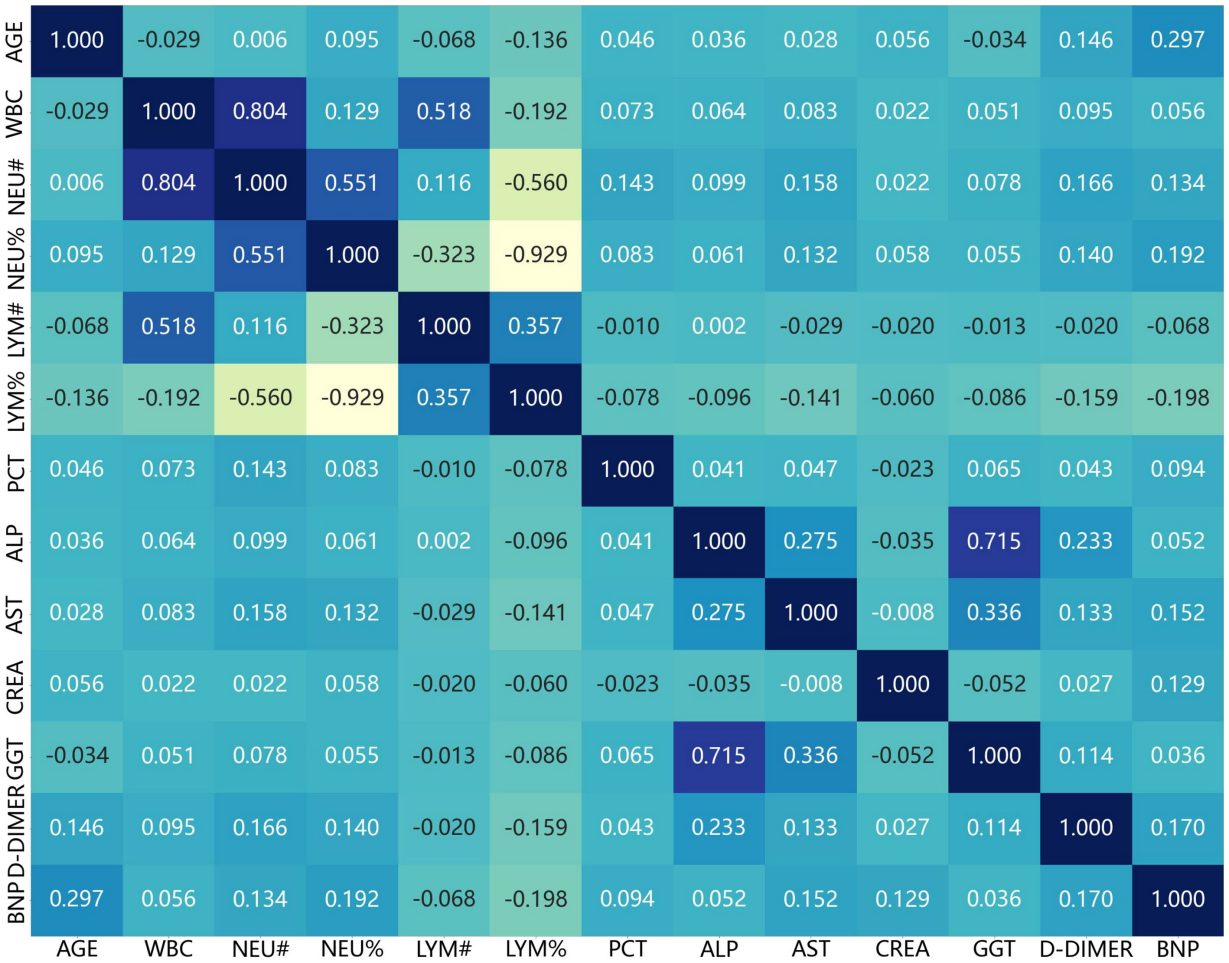
Path diagram of LASSO regression variable coefficient.

A significant negative correlation is prominently observed between the neutrophil percentage (NEU%) and the lymphocyte percentage (LYM%) (*r* = − 0.72, *p* < 0.01), as indicated by the deep blue color in the corresponding cell of the heatmap. This inverse relationship aligns well with the physiological knowledge of the immune system (21). During immune responses, neutrophils and lymphocytes typically display reciprocal fluctuations. For example, in specific inflammatory scenarios related to acute exacerbation of chronic obstructive pulmonary disease (AECOPD), an elevation in neutrophil levels might be accompanied by a reduction in lymphocyte levels, and conversely. This relationship plays a crucial role in assessing the immune status of AECOPD patients and could have far-reaching implications for understanding disease progression and predicting prognosis. Certain variables exhibit moderate positive correlations. For instance, the light red-shaded cells in the heatmap signify positive correlations among variables such as the white blood cell count (WBC), neutrophil count (NEU#), and NEU%. This correlation is biologically plausible since neutrophils are a major constituent of white blood cells. An increase in the total white blood cell count often stems from an increase in neutrophil count, which is manifested as the positive correlation seen in the heatmap. Such associations imply that these variables might be governed by common underlying physiological mechanisms or influenced by similar factors in AECOPD patients. The majority of cells in the heatmap are characterized by light shades, which denote weak correlations among most of the remaining variables. This suggests that, in general, these variables may operate relatively independently or that their relationships are intricate and not easily discernible through simple linear correlations. For example, variables such as age (AGE), and various biochemical markers such as alkaline phosphatase (ALP) and aspartate aminotransferase (AST) demonstrate weak correlations with many other variables. This information is of great significance for building predictive models as it indicates that each of these variables may contribute unique information rather than being redundant to one another (Figure 2).

**Figure 3:**
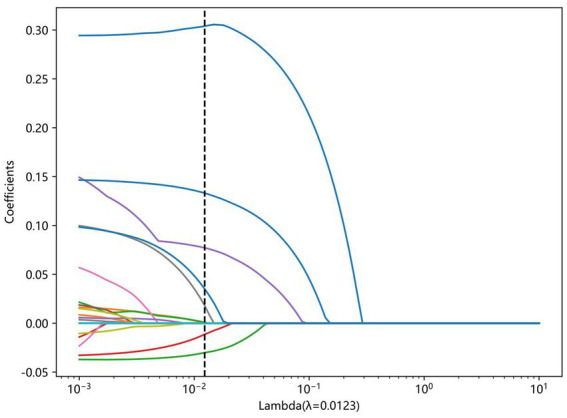
Path diagram of LASSO regression variable coefficients.

**Figure 4:**
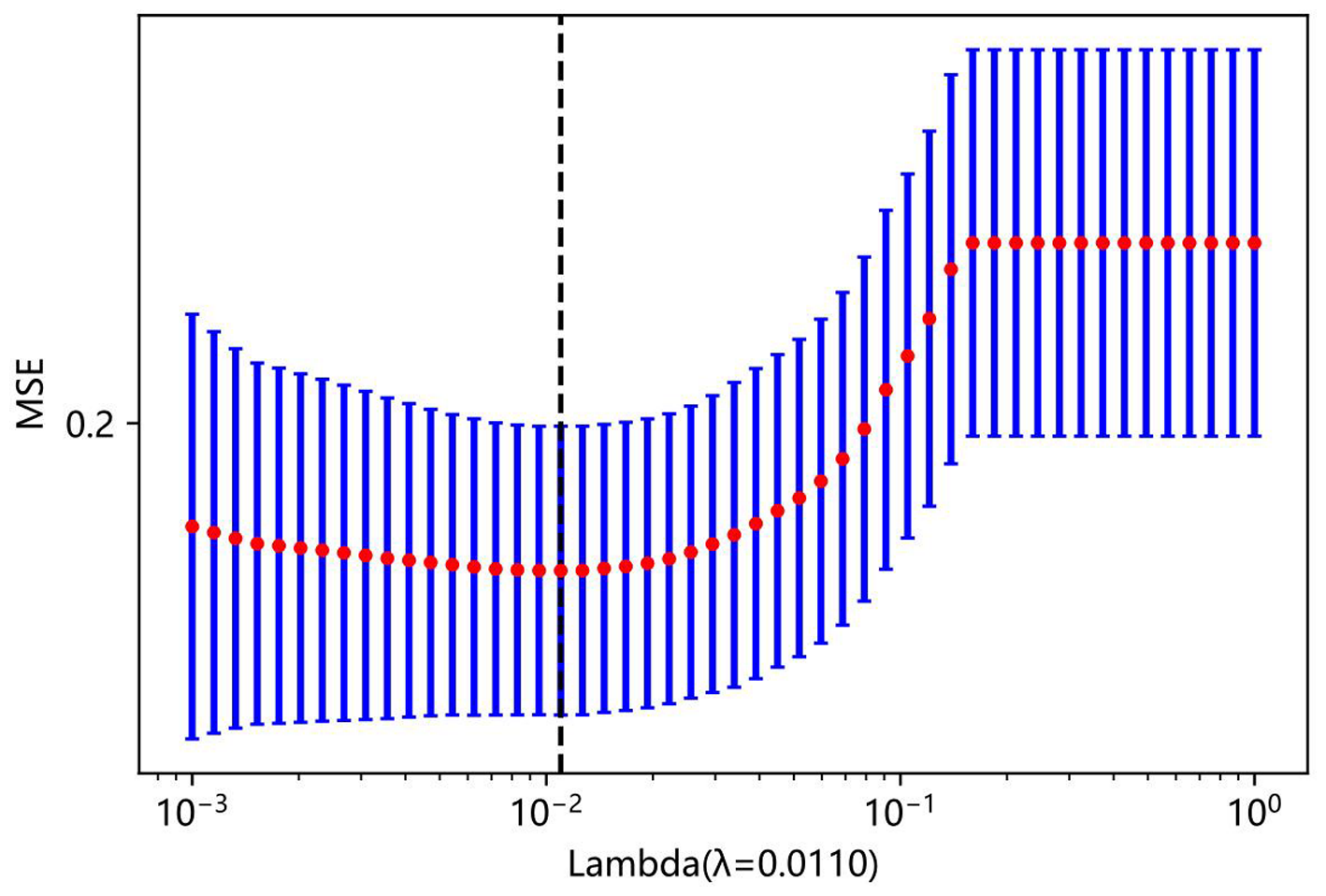
LASSO model error.

**Figure 5:**
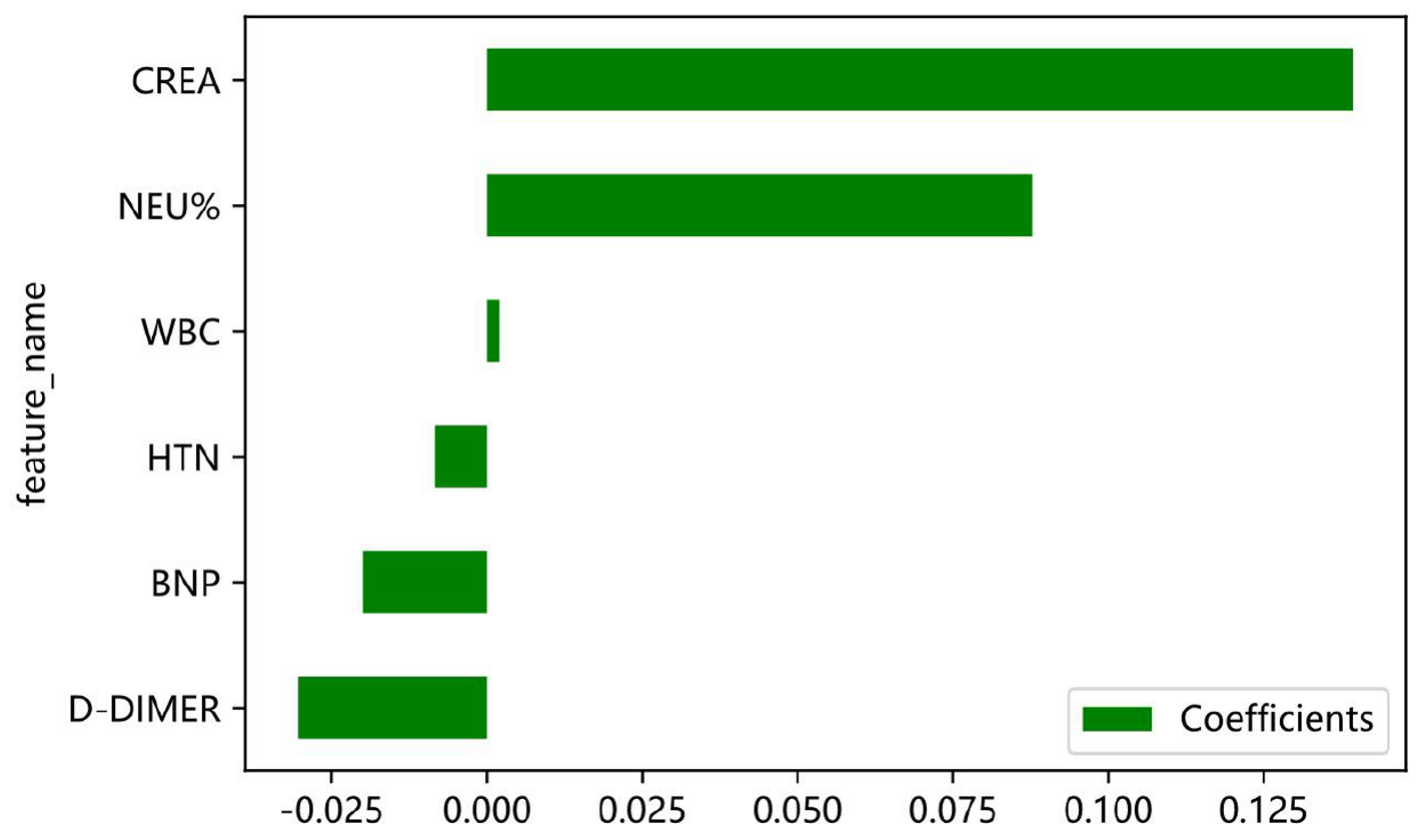
LASSO model coefficient weights.

LASSO regression was used to select relevant features from the dataset. The penalty coefficient corresponding to the minimum error was 0.01. Six variables closely related to AECOPD are WBC, NEU%, CREA, D-dimer, BNP, and HTN, and the corresponding LASSO weight coefficients are shown in Figure 3. The LASSO analysis was conducted using a 10-fold cross-validation method. The LASSO model error plot is shown in Figure 4. The coefficient path plot is shown in Figure 5. As shown in Equation 6, the LASSO regression equation retained six core predictive variables through shrinkage selection. Among them, CREA showed the strongest positive predictive contribution to the need for treatment escalation, with a coefficient of 0.139369. In contrast, HTN exerted the weakest negative effect, with a coefficient of −0.008358. Positive variables, including WBC, NEU%, and CREA, indicated that elevated levels of these indicators were associated with an increased risk of treatment escalation. Negative variables, including D-dimer, BNP, and HTN, suggested that higher levels or comorbidity with these conditions were related to a decreased predicted risk. The sign and magnitude of the model coefficients intuitively reflected the direction and magnitude of the impact of each clinical indicator on the decision regarding treatment escalation in hospitalized AECOPD patients.


$$\begin{array}{l} \hat{Y}=0.328+0.00193\cdot \mathrm{wBc}+0.0878\cdot \mathrm{NEu}\%+0.1394\cdot \text{cREA}\\ -0.0304\cdot D-\text{DIMER}‐0.0200\cdot \mathrm{BNP}-0.00836\cdot \mathrm{HTN} \end{array}$$ (6)

Multivariate logistic regression analysis was performed in this study, and the results are summarized in Table 2. Table 2 shows that among the six core variables, three were significantly associated with the need for treatment escalation (*p* < 0.05), while the remaining three variables had no significant association (*p* > 0.05). The regression coefficient (*β*) of NEU% (neutrophil percentage) was 0.592, with an OR of 1.807 (95% CI: 1.558–2.096, *p* < 0.001); the *β* value of CREA (creatinine) was 0.702, with an OR of 2.018 (95% CI: 1.787–2.280, p < 0.001). The β value of D-dimer (D-dimer) was −0.558, with an OR of 0.573 (95% CI: 0.439–0.747, p < 0.001); the β value of HTN (hypertension) was −0.303, with an OR of 0.739 (95% CI: 0.582–0.937, *p* = 0.013). The β value of WBC (white blood cell count) was −0.164, with an OR of 0.849 (95% CI: 0.638–1.130, *p* = 0.261); the β value of BNP (B-type natriuretic peptide) was −0.1, with an OR of 0.905 (95% CI: 0.797–1.028, p = 0. 123).

**Table 2:** Multivariate logistic regression result interpretation.

Variable	Coefficient (β)	OR (95% CI)	*p*-value
WBC	−0.164	0.849 (0.638–1. 130)	0.261
NEU%	0.592	1.807 (1.558–2.096)	<0.1
CREA	0.702	2.018 (1.787–2.280)	<0.1
D-dimer	−0.558	0.573 (0.439–0.747)	<0.1
BNP	−0. 1	0.905 (0.797–1.028)	0. 123
HTN	−0.303	0.739 (0.582–0.937)	0.013

### 3.3 Model performance

All models were built and optimized for parameters using the training set, and their generalization performance was validated on an independent test set. Table 3 presents the complete performance metrics of the six models on both the training and test sets. Among them, the Random Forest (RandomForest) model performed best on the training set, while the extreme gradient boosting (XGBoost) model showed more stable and excellent performance on the test set. In the training set, the area under the curve (AUC) of the Random Forest model reached 0.995 [95% confidence interval (CI): 0.991–0.998], and its accuracy, sensitivity, specificity, positive predictive value (PPV), and negative predictive value (NPV) were 0.978, 0.984, 0.975, 0.951, and 0.992, respectively, with all indicators leading among the six models. The XGBoost model also demonstrated strong training performance, with an AUC of 0.960 (95% CI: 0.940–0.980), and its accuracy, sensitivity, specificity, PPV, and NPV were 0.917, 0.869, 0.941, 0.880, and 0.935, respectively. The remaining models showed moderate predictive capabilities on the training set, with AUC ranked from high to low as follows: K-nearest neighbors (KNN) (AUC = 0.891, 95% CI: 0.876–0.907), support vector machine (SVM) (AUC = 0.838, 95% CI: 0.814–0.862), logistic regression (LR) (AUC = 0.758, 95% CI: 0.732–0.784), and Naive Bayes (AUC = 0.712, 95% CI: 0.683–0.742). The performance validation results on the test set showed that the AUC of each model was generally lower than that of the training set, but the overall predictive efficacy remained acceptable. The XGBoost model demonstrated the best comprehensive performance on the test set, with an AUC of 0.824 (95% CI: 0.804–0.844), and its accuracy, sensitivity, specificity, PPV, and NPV were 0.805, 0.650, 0.872, 0.669, and 0.859, respectively. The Random Forest model’s AUC decreased to 0.795 (95% CI: 0.753–0.836) on the test set, indicating a certain degree of overfitting, with its accuracy (0.754) and PPV (0.562) at a relatively lower level among the models. The remaining models still maintained reasonable predictive capabilities on the test set, with AUC ranked from high to low as follows: SVM (AUC = 0.790, 95% CI: 0.7465–0.8335), LR (AUC = 0.763, 95% CI: 0.722–0.805), KNN (AUC = 0.750, 95% CI: 0.7062–0.7936), and Naive Bayes (AUC = 0.714, 95% CI: 0.666–0.762).

**Table 3:** Performance metrics of machine learning models.

Model	Dataset	Accuracy	AUC (95% CI)	Sensitivity	Specificity	PPV	NPV
LR	train	0.697	0.758 (0.732–0.784)	0.751	0.67	0.531	0.844
LR	test	0.78	0.763 (0.722–0.805)	0.552	0.873	0.639	0.827
Naive Bayes	train	0.699	0.712 (0.683–0.742)	0.63	0.733	0.54	0.799
Naive Bayes	test	0.765	0.714 (0.666–0.762)	0.557	0.85	0.604	0.825
SVM	train	0.831	0.838 (0.814–0.862)	0.652	0.92	0.802	0.842
SVM	test	0.781	0.79 (0.7465–0.8335)	0.656	0.834	0.615	0.856
KNN	train	0.791	0.891 (0.876–0.907)	0.812	0.781	0.649	0.893
KNN	test	0.792	0.75 (0.706–0.793)	0.475	0.934	0.713	0.811
RandomForest	train	0.978	0.995 (0.991–0.998)	0.984	0.975	0.951	0.992
RandomForest	test	0.754	0.795 (0.753–0.836)	0.689	0.788	0.562	0.86
XGBoost	train	0.917	0.960 (0.940–0.980)	0.869	0.941	0.88	0.935
XGBoost	test	0.805	0.824 (0.804–0.844)	0.65	0.872	0.669	0.859

Figure 3 shows the receiver operating characteristic (ROC) curves of the six models on the test set, and Figure 4 is the ROC curves of the XGBoost model on the training and test sets.

The confusion matrix of the XGBoost model is shown in Figure 5 (Model: XGBoost). In this matrix, the model achieved 173 true negatives (patients correctly identified as non-AECOPD), 138 true positives (patients correctly identified as AECOPD), 63 false positives (patients incorrectly identified as AECOPD), and 78 false negatives (patients incorrectly identified as non-AECOPD).

**Figure 6:**
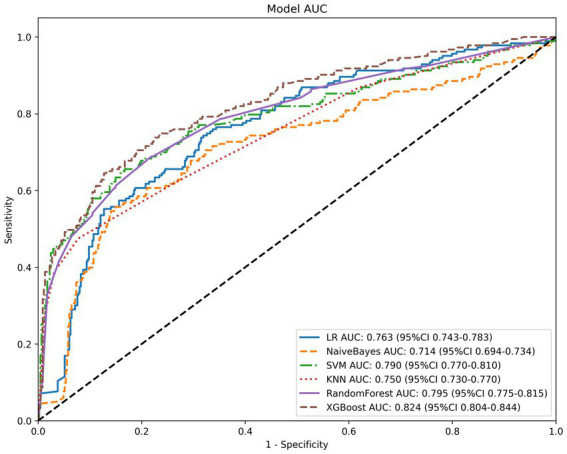
ROC curves of all models.

**Figure 7:**
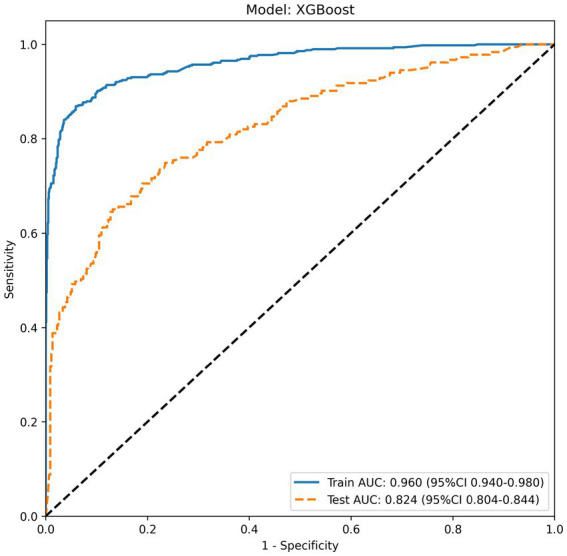
ROC curves of XGBoost models.

**Figure 8:**
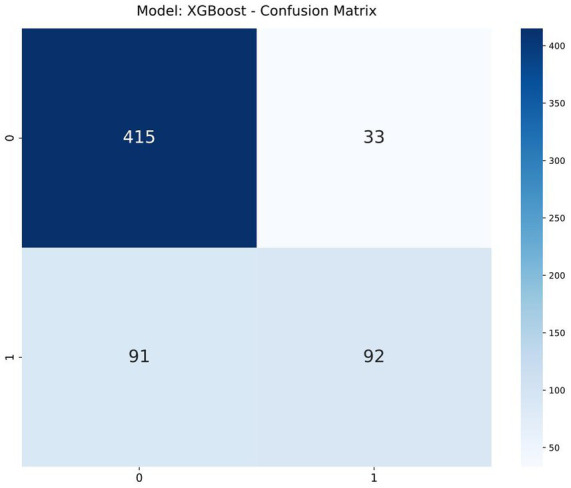
Confusion matrix of XGBoost.

To evaluate the clinical utility of the XGBoost model in the diagnosis of AECOPD, we performed a decision curve analysis (DCA), as shown in Figure 6 (Model XGBoost DCA). The x-axis represents the threshold probability, which denotes the minimum probability at which a clinician would opt for intervention (e.g., initiating treatment for suspected AECOPD). The y-axis indicates the net benefit, calculated as the difference between the benefits of correct interventions and the harms of unnecessary interventions, normalized by the number of patients. The curve labeled “Model” corresponds to the XGBoost model. It demonstrates that within a specific range of threshold probabilities, the model yields a positive net benefit, implying that guiding diagnostic decisions for AECOPD with this model in this interval brings more clinical benefits than the two extreme strategies of “treating all” (Treat all) and “treating none” (Treat none) (Figures 7, 8).

**Figure 9:**
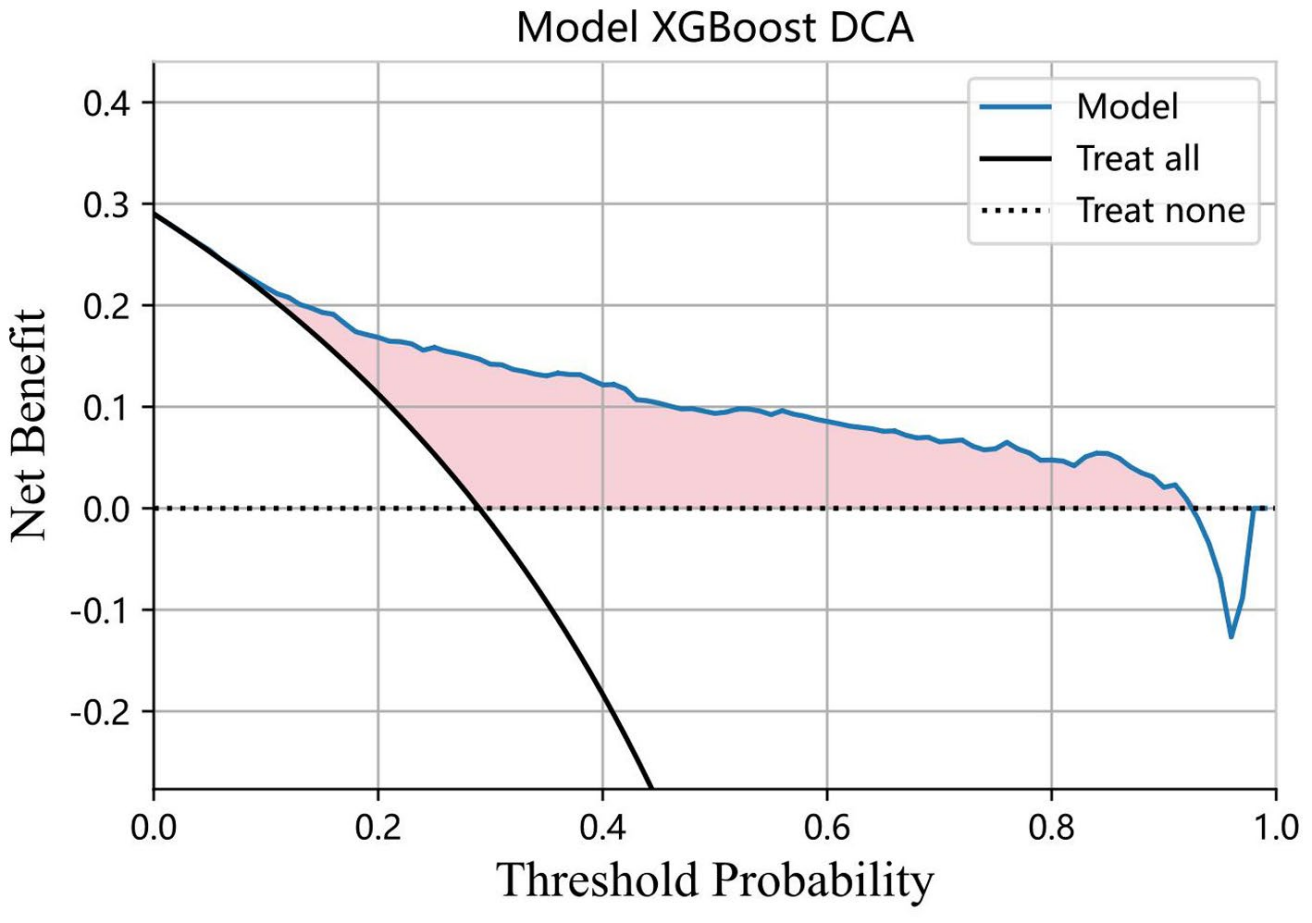
DCA curves of XGBoost models.

**Figure 10:**
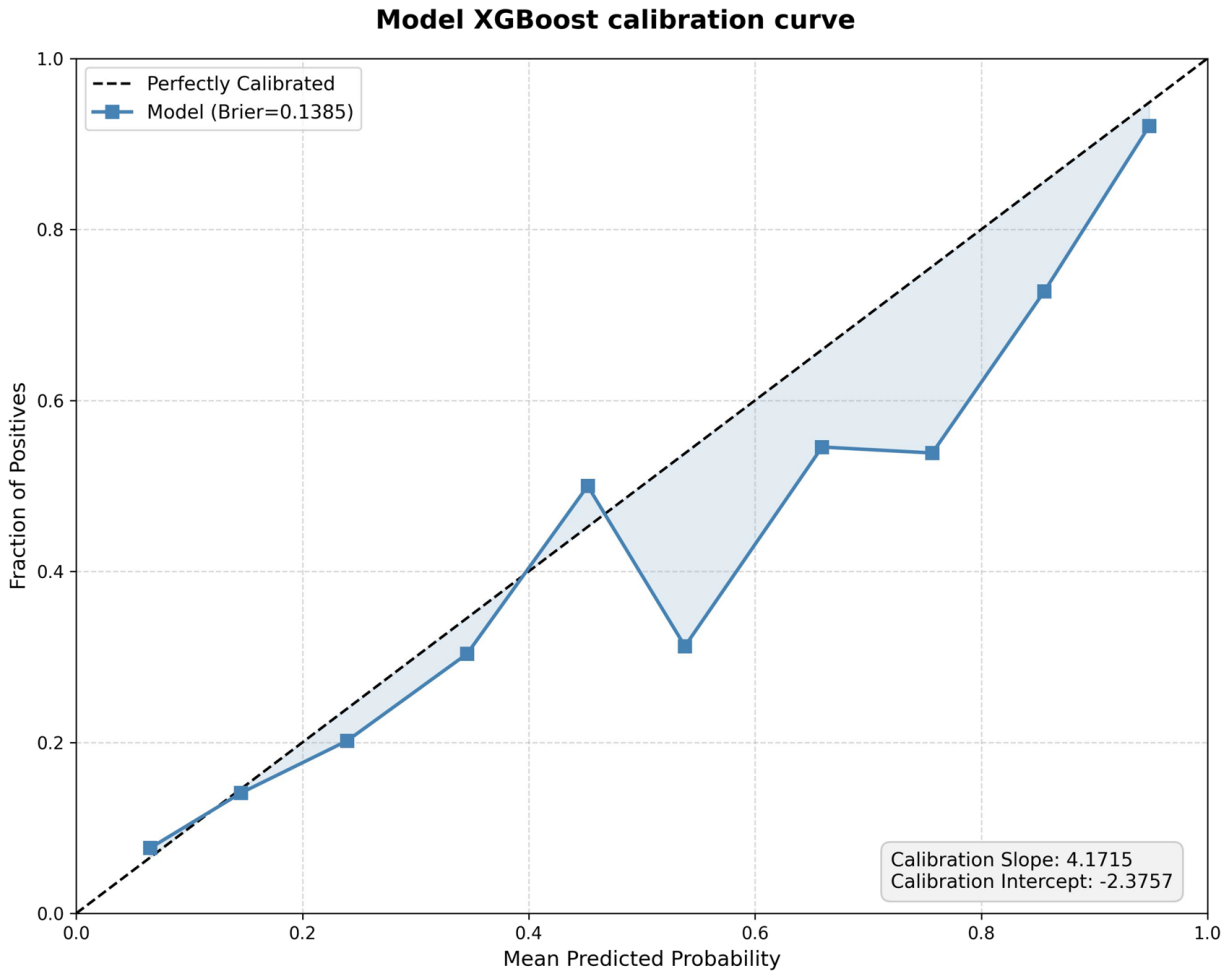
Calibration curve of XGBoost models.

The calibration curve of the XGBoost model is presented in Figure 6. The model’s predicted probabilities were plotted against the observed fraction of positives across risk strata, with the dashed line representing perfect calibration. The Brier score of the model was 0.1385. The calibration slope was 4.1715, and the calibration intercept was −2.3757, indicating the degree of deviation from ideal calibration (Figures 9, 10).

### 3.4 Interpretability analysis

**Figure 11:**
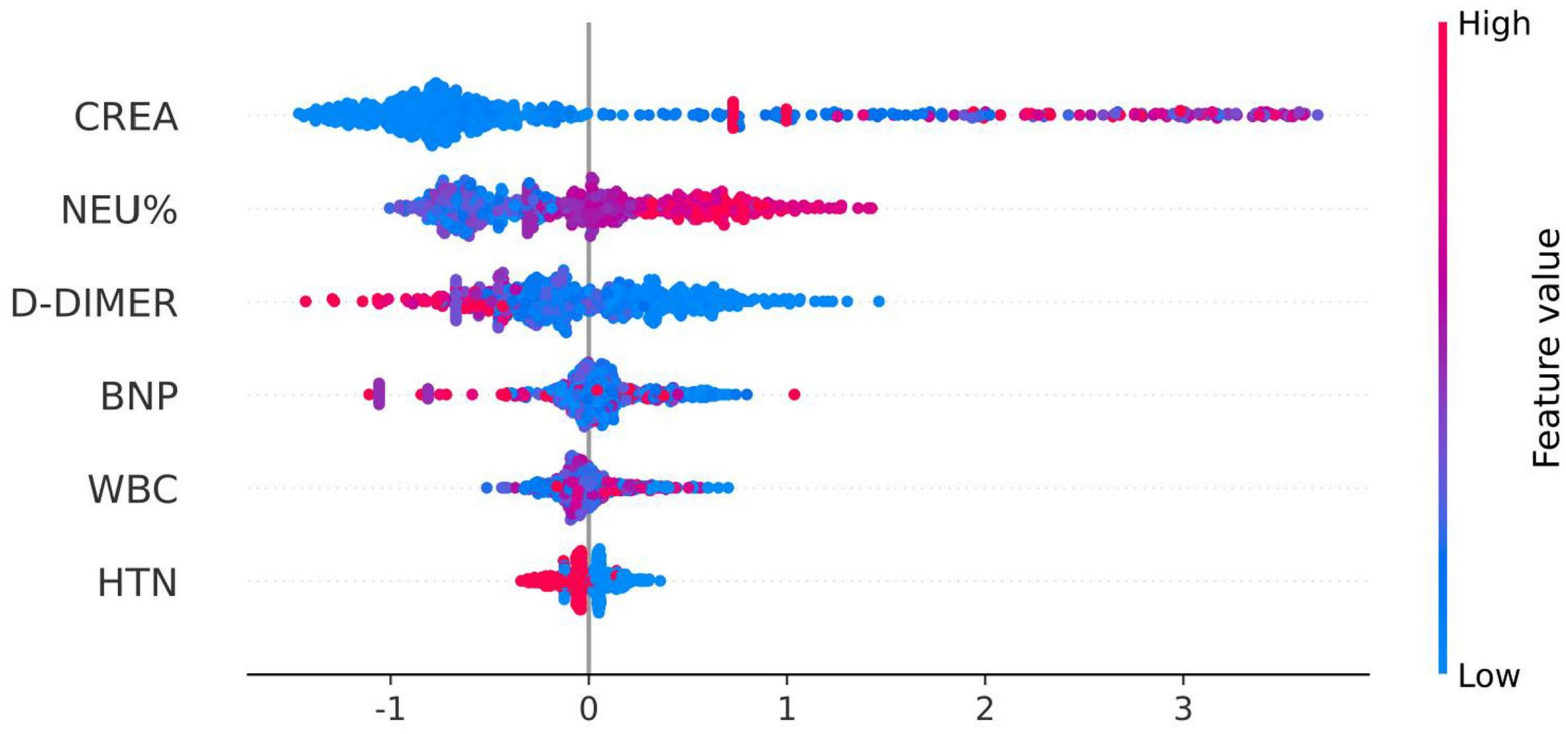
SHAP value (impact on model output).

To elucidate the importance and impact of each feature on the XGBoost model for predicting AECOPD, We plotted a SHAP swarm plot, as shown in Figure 11. In this plot, the horizontal axis represents the SHAP value, which quantifies the magnitude and direction of a feature’s impact on the model output. The vertical axis lists the features sorted by their overall influence on the model. Each data point corresponds to a specific instance of a feature, with the color gradient (from blue to red) indicating the feature value (low to high). Creatinine (CREA), neutrophil percentage (NEU%), D-Dimer, brain natriuretic peptide (BNP), white blood cell (WBC) count, and hypertension (HTN) are six important factors for distinguishing AECOPD.

**Figure 12:**
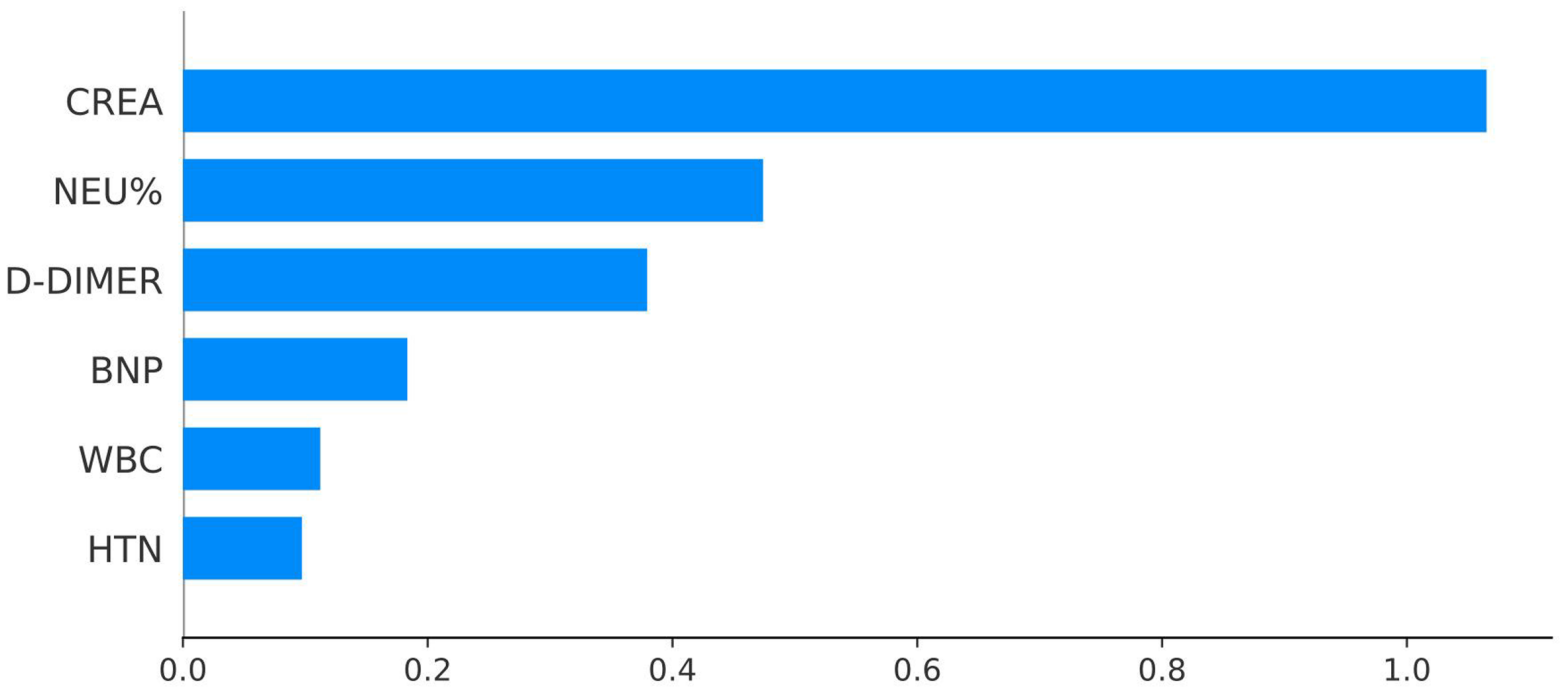
Feature’s impact on the model output.

The feature importance in the XGBoost model for AECOPD prediction is clarified by the SHAP weight plot, as presented in Figure 12. In this study, the horizontal axis represents the mean absolute SHAP value, which quantifies the average magnitude of a feature’s impact on the model output. The vertical axis lists the features (including CREA, NEU%, D-Dimer, BNP, WBC, and HTN) sorted by their mean absolute SHAP value from highest to lowest.

**Figure 13:**
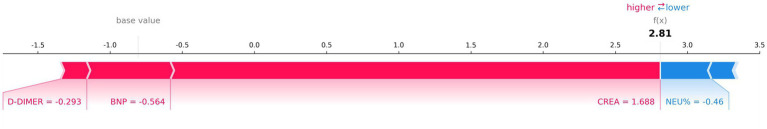
Prediction process of the XGBoost model for AECOPD risk.

Figure 13 illustrates the prediction process of the XGBoost model for AECOPD risk. The base value in the plot represents the average model output across all samples. The contribution of each feature is depicted as a segment, where red segments indicate positive impacts (increasing the predicted risk) and blue segments indicate negative impacts (decreasing the predicted risk). For this specific patient, the model predicted a probability of 0.943 for AECOPD (Class 1). Key feature values include NEU% = − 0.46, CREA = 1.688, BNP = -0.564, and D-Dimer = −0.293. These feature contributions collectively deviate from the base value, resulting in a final model output (f(x) = 2.81) that corresponds to a high predicted probability of AECOPD.

### 3.5 Application of the model

To enhance the applicability of the XGBoost model in this study, we further developed a web-based application that rapidly predicts a patient’s risk of acute exacerbation of COPD (AECOPD) from six core clinical and laboratory indicators entered via a web interface. The six core indicators are creatinine (CREA), neutrophil percentage (NEU%), D-dimer (D-dimer), B-type natriuretic peptide (BNP), white blood cell count (WBC), and hypertension (HTN). To improve the model’s accessibility and translational value in clinical research, the optimized XGBoost model was deployed on a dedicated web platform (http://121.5.46.90:1002/). The interface is easy to operate: Clinicians need only to enter the patient’s admission-day values for the six indicators to obtain an immediate model prediction. This tool is intended as a research-assistive prototype, has not undergone large-scale external validation, and is not intended for use in clinical decision-making.

## 4 Discussion

This study developed and validated multiple machine learning models to predict the risk of treatment escalation among hospitalized patients with acute exacerbation of chronic obstructive pulmonary disease (AECOPD). Among the six algorithms evaluated, the XGBoost model demonstrated the optimal predictive performance, achieving an area under the receiver operating characteristic curve (AUC) of 0.824 (95% CI: 0.804–0.844) on the independent test set, with an accuracy of 0.805, specificity of 0.872, and negative predictive value of 0.859. These findings suggest that machine learning approaches, particularly tree-based ensemble methods, can effectively leverage routinely collected clinical and laboratory data to identify patients at risk of clinical deterioration during hospitalization.

### 4.1 Model performance and comparison

The comparative analysis of the six machine learning models revealed important insights regarding their generalization capabilities. On the training set, the Random Forest model achieved near-perfect performance (AUC = 0.995, 95% CI: 0.991–0.998), substantially outperforming all other algorithms. However, its test set AUC dropped to 0.795 (95% CI: 0.753–0.836), representing a decline of 0.200 and indicating considerable overfitting. This pattern—excellent training performance but diminished test set generalization—was also observed in the KNN model, whose AUC decreased from 0.891 (95% CI: 0.876–0.907) in training to 0.750 (95% CI: 0.706–0.794) in testing. In contrast, the XGBoost model demonstrated superior stability, with a training AUC of 0.960 (95% CI: 0.940–0.980) and test AUC of 0.824 (95% CI: 0.804–0.844), a performance decrement of only 0.136. This smaller generalization gap highlights the effectiveness of XGBoost’s built-in regularization mechanisms, including shrinkage and column subsampling, which help mitigate overfitting while preserving the model’s ability to capture complex non-linear relationships in the data (22). The support vector machine (SVM) and logistic regression (LR) models exhibited more modest but stable performance, with test AUCs of 0.790 (95% CI: 0.747–0.834) and 0.763 (95% CI: 0.722–0.805), respectively, and minimal overfitting. The superior performance of XGBoost compared to traditional logistic regression (AUC = 0.763) aligns with a growing body of evidence demonstrating the advantages of ensemble machine learning methods for clinical risk prediction (16, 23). Unlike logistic regression, which assumes linear relationships and independence among predictors, tree-based models can automatically capture interactions and non-linear effects without explicit specification. This flexibility is particularly valuable in complex clinical scenarios such as AECOPD, where pathophysiological processes involve intricate interplay among inflammatory markers, organ function indicators, and comorbid conditions (30).

### 4.2 Key risk factors and clinical interpretation

The integration of LASSO regression for initial feature selection and SHAP analysis for model interpretability provided complementary insights into the factors driving AECOPD risk. LASSO regression identified six core predictors—creatinine (CREA), neutrophil percentage (NEU%), D-dimer, brain natriuretic peptide (BNP), white blood cell count (WBC), and hypertension (HTN)—with CREA demonstrating the strongest positive coefficient (*β* = 0. 1,394) in the linear framework. Multivariate logistic regression further confirmed the independent associations of NEU% (OR = 1.807, 95% CI: 1.558–2.096, *p* < 0.001), CREA (OR = 2.018, 95% CI: 1.787–2.280, *p* < 0.001), D-dimer (OR = 0.573, 95% CI: 0.439–0.747, *p* < 0.001), and HTN (OR = 0.739, 95% CI: 0.582–0.937, *p* = 0.013) with the need for treatment escalation. SHAP analysis of the final XGBoost model revealed that CREA was the most influential predictor, followed by NEU%, D-dimer, BNP, WBC, and HTN (Figure 12). This ordering differs from the LASSO coefficient magnitudes, reflecting the ability of the non-linear XGBoost model to capture more complex relationships that linear methods cannot adequately represent. The prominence of CREA—a marker of renal function—as the top predictor warrants particular attention. Elevated creatinine may reflect prerenal azotemia secondary to decreased oral intake, increased insensible losses, or reduced cardiac output during severe exacerbations or may indicate underlying chronic kidney disease as a comorbid condition. Regardless of the mechanism, the strong association between renal dysfunction and need for treatment escalation suggests that AECOPD patients with elevated creatinine warrant closer monitoring and more aggressive early intervention. The identification of NEU% as the second most important predictor aligns with the well-established role of neutrophilic inflammation in AECOPD pathogenesis (1, 24). Acute exacerbations are characterized by enhanced airway inflammation, with neutrophils playing a central role through the release of proteases and oxidative burst (26, 31, 32). The positive association between NEU% and treatment escalation risk (OR = 1.807 in logistic regression) supports the clinical utility of this routine laboratory marker for early risk stratification. Furthermore, the significant negative correlation between NEU% and lymphocyte percentage (LYM%) observed in the correlation analysis (r = −0.72, *p* < 0.01) underscores the dynamic interplay between innate and adaptive immune responses during acute exacerbations, suggesting that a comprehensive assessment of immune status may provide additional prognostic information (21, 29). The inverse associations observed for D-dimer and BNP require careful interpretation. While elevated D-dimer typically indicates activation of coagulation and fibrinolysis, and elevated BNP suggests cardiac strain, both showed negative coefficients in the LASSO model and protective effects in multivariate analysis. This counterintuitive finding may reflect the complexity of the study population or potential confounding by indication. Alternatively, it is possible that patients with markedly elevated D-dimer or BNP were more likely to receive alternative diagnoses (such as pulmonary embolism or acute heart failure) and thus were managed differently although this cannot be confirmed from the available data. Further research with more detailed phenotyping is needed to elucidate these relationships. The inclusion of hypertension as a predictor, albeit with relatively modest influence, highlights the importance of cardiovascular comorbidities in COPD outcomes. Patients with hypertension may have different patterns of medication use, cardiovascular reserve, or susceptibility to fluid overload during exacerbations, all of which could influence the need for treatment escalation. The negative association (OR = 0.739) suggests that hypertensive patients in this cohort may have been paradoxically at lower risk, possibly due to more frequent healthcare contact, better medication adherence, or protective effects of certain antihypertensive agents. This finding merits investigation in future studies.

### 4.3 Clinical implications and model utility

From a clinical practice perspective, the XGBoost model developed in this study offers several potential applications. First, all six predictive variables are routinely collected within 24 h of hospital admission as part of standard clinical care, enabling risk prediction without additional testing or patient burden. Second, the model’s high negative predictive value (NPV = 0.859) indicates that it can reliably identify patients at low risk of requiring treatment escalation, potentially supporting decisions to step down monitoring intensity or expedite discharge planning. Third, the integration of SHAP analysis enhances model transparency, allowing clinicians to understand which factors contributed most strongly to an individual patient’s predicted risk (Figure 13). The temporal design of this study—with predictor variables collected prior to the outcome—supports the model’s use for early risk stratification. By identifying high-risk patients soon after admission, clinicians can prioritize resources, intensify monitoring, and consider preventive interventions such as early initiation of non-invasive ventilation, closer fluid management, or consultation with critical care teams. For patients identified as low-risk, the model may support more efficient resource allocation and reduced length of stay (25). The development of a web-based application (http://121.5.46.90:1002/) represents an important step toward translational implementation. By providing immediate risk predictions based on six easily obtained variables, this tool facilitates bedside risk assessment without requiring local installation or integration with electronic health record systems. However, it is essential to emphasize that this application is intended as a research-assistive prototype and has not undergone prospective validation. It should not be used for clinical decision-making without further external validation and regulatory approval.

### 4.4 Strengths and limitations

This study possesses several notable strengths. The use of temporally split validation (training on earlier patients and testing on later patients) better reflects real-world model deployment scenarios than random cross-validation as it evaluates model performance on future patients. Additionally, the integration of LASSO for feature selection, multiple algorithms for model development, and SHAP for interpretability provides a comprehensive analytical framework, while the focus on routinely available clinical variables enhances the model’s potential for broad implementation. However, several limitations must be acknowledged. First, the single-center design and retrospective nature may limit generalizability and introduce potential confounding, despite efforts to standardize data collection. Multi-center external validation is essential before broader application. Second, although the predictor set is clinically relevant, it excludes potentially important variables such as arterial blood gas measurements, prior exacerbation history, or detailed pulmonary function tests (27, 34). Third, despite the model’s satisfactory discriminative ability, it exhibited suboptimal calibration (calibration slope = 4.1715, intercept = −2.3757), indicating that predicted probabilities should not be interpreted as accurate absolute risk estimates without recalibration. Fourth, the model has not undergone prospective validation, and its impact on clinical decision-making and patient outcomes remains unknown, although decision curve analysis demonstrated positive net benefit across a range of threshold probabilities.

## 5 Conclusion

This study demonstrates that the XGBoost machine learning model, using six routinely collected clinical and laboratory variables, can effectively predict the need for treatment escalation in hospitalized COPD patients. The model achieved an AUC of 0.824 on an independent test set, outperforming other algorithms including logistic regression and random forest. SHAP analysis identified creatinine, neutrophil percentage, D-dimer, BNP, white blood cell count, and hypertension as the most influential predictors, with creatinine emerging as the dominant factor. The model’s high negative predictive value supports its potential utility for identifying low-risk patients, while its calibration limitations highlight the need for recalibration before absolute risk interpretation. External validation and prospective studies are necessary before clinical implementation. With further refinement and validation, this model could contribute to improved risk stratification, more efficient resource allocation, and ultimately better outcomes for patients with COPD.

## Data Availability

The original contributions presented in the study are included in the article/Supplementary material, further inquiries can be directed to the corresponding author.
